# A stable XPG protein is required for proper ribosome biogenesis: Insights on the phenotype of combinate *Xeroderma Pigmentosum*/Cockayne Syndrome patients

**DOI:** 10.1371/journal.pone.0271246

**Published:** 2022-07-08

**Authors:** Florent TAUPELET, Lise-Marie DONNIO, Charlène MAGNANI, Pierre-Olivier MARI, Giuseppina GIGLIA-MARI

**Affiliations:** Institut NeuroMyoGène–Physiopathologie et Génétique du Neurone et du Muscle, CNRS UMR 5261, INSERM U1315, Faculté de Médecine et de Pharmacie Rockefeller, Université Claude Bernard Lyon 1, Lyon, France; University of Toronto, CANADA

## Abstract

Nucleotide Excision Repair is one of the five DNA repair systems. More than 30 proteins are involved in this process, including the seven XP proteins. When mutated, the genes coding for these proteins are provoking the rare disease *Xeroderma Pigmentosum*, which causes cutaneous defects and a high prevalence of skin cancers in patients. The CSA and CSB proteins are also involved in Nucleotide Excision Repair, and their mutation leads to Cockayne Syndrome, another rare disease, causing dwarfism, neurodegeneration, and ultimately early death, but without high skin cancer incidence. Some mutations of *ERCC5*, the gene coding for XPG, may give rise to a combined *Xeroderma Pigmentosum* and Cockayne Syndrome. A defect in Nucleotide Excision Repair alone cannot explain all these phenotypes. XPG has been located in the nucleolus, where ribosome biogenesis happens. This energy-consuming process starts with the transcription of the ribosomal DNA in a long ribosomal RNA, the pre-rRNA 47S, by RNA Polymerase 1. 47S pre-rRNA undergoes several cleavages and modifications to form three mature products: the ribosomal RNAs 18S, 5.8S and 28S. In the cytoplasm, these three products will enter the ribosomes’ composition, the producers of protein in our cells. Our work aimed to observe ribosome biogenesis in presence of an unstable XPG protein. By working on *Xeroderma Pigmentosum/*Cockayne Syndrome cell lines, meaning in the absence of XPG, we uncovered that the binding of UBF, as well as the number of unresolved R-loops, is increased along the ribosomal DNA gene body and flanking regions. Furthermore, ribosomal RNA maturation is impaired, with increased use of alternative pathways of maturation as well as an increase of immature precursors. These defective processes may explain the neurodegeneration observed when the XPG protein is heavily truncated, as ribosomal homeostasis and R-loops resolution are critical for proper neuronal development.

## Introduction

Across evolution, maintaining DNA integrity has always been of utmost importance. Because this integrity is constantly challenged by several genotoxic threats, cells developed a toolbox of repair mechanisms to ensure DNA preservation [1]. These repair mechanisms are greatly specialized, and a given type of damage will be repaired by a specific repair mechanism. To repair helix-distorting lesions, the cell relies on Nucleotide Excision Repair (NER), which is divided into two sub-pathways: the global genome repair (GG-NER), in charge of keeping the whole genome free from this type of lesions, and the transcription-coupled repair (TC-NER), which repairs the lesion where the actively transcribing RNA Polymerase 2 is stalled [2] or, as it has been recently discovered, the ribosomal genes, transcribed by RNA Polymerase 1 (RNAP1) [3].

NER mechanism requires the action of more than 30 proteins [1]. Impairment of the NER system can cause various diseases such as: Trichothiodystrophy (TTD) [4], Cockayne syndrome (CS) and *Xeroderma Pigmentosum* (XP) [5]. Xeroderma Pigmentosum disease is caused by mutations in genes coding for XP proteins. XP patients suffer from extreme photosensitivity and the incidence of skin cancer is dramatically increased in these patients, presenting over 10000 fold risk of developing non-melanocytic skin cancers and 2000 fold risk of developing melanoma compared to the healthy population [6]. Furthermore, these cancers appear at a very young age (2–5 years).

CS is caused by a mutation of the *ERCC8* or *ERCC6* gene, coding respectively for the CSA and CSB proteins. Both of them are involved in the TC-NER, associating with the stalled polymerase at the site of the damage [7]. CS patients have specific wizened facies, with sunken eyes [8], mental retardation due to neuronal loss, hearing loss [9], cachectic dwarfism, and most importantly a poor prognosis, with an average life-span of 5–10 years. However, contrary to XP patients, they do not suffer from a high cancer incidence [10]. Nevertheless, patients with specific mutations of the genes coding for XPB or XPD, two proteins of the TFIIH complex, which has several roles in NER and beyond [11], or XPG, may develop a combination of *Xeroderma Pigmentosum* and Cockayne Syndrome (XP/CS), that associates the severe photosensitivity and the high skin cancer incidence to the neurologic and somatic manifestations of CS patients, worsening even more the prognosis of the patients [12].

The XPG protein is a 3’ endonuclease, coded by the *ERCC5* gene. In NER, XPG is in charge of the cleavage in 3’ of the lesion. However, this protein plays several other roles: it interacts with an elongating RNAP2 [13], it is involved in Base Excision Repair [14, 15] and it is contributing to the resolution of R-loops, RNA: DNA hybrid structures that tends to accumulate in actively transcribed regions, in a mechanism called Transcription Associated Homologous Recombination Repair (TA-HRR) [16]. Mutations in the *ERCC5* gene can give rise to either an XP phenotype (XP-G) or a more severe syndrome that combines both features of XP and features of CS (XP-G/CS). One of the hypotheses to explain this fact is the extent of the mutation: a missense point mutation of XPG could cause only XP-G, while nonsense mutations, leading to the production of truncated proteins, could lead to a combination of the two diseases [17].

Intriguingly, a fraction of the XPG protein is localized within the nucleolus and colocalize with the CSB, TFIIH and transcribing RNAP1 complex, hinting at a possible role in ribosome biogenesis [18]. Ribosome biogenesis starts with the transcription of ribosomal DNA (rDNA) by RNAP1 and takes place within the nucleolus. RNAP1 transcription represents the majority of the transcriptional activity of a cell in certain cases (60% in yeast [19]).The product of RNAP1 transcription is a long ribosomal RNA (rRNA), the 47S pre-rRNA. This first transcription product will undergo several cleavages, degradations, post-transcriptional modifications such as methylations, and exports to finally produce three mature rRNAS: the 18S, the 5.8S and the 28S. In association with the 5S RNA, transcribed by the RNA Polymerase 3 (RNAP3), these three rRNAs will enter in the composition of ribosomes. A ribosome is made of two subunits: the large subunit (LSU), constituted by the 5S, 5.8S and 28S rRNAs associated with 49 riboproteins, and the small one (SSU), constituted by the 18S rRNAs associated with 33 riboproteins [20]. Furthermore, the deregulation of the transcriptional activity of RNAP1 has previously been linked with neurodegenerative symptoms [21] and ribosome biogenesis has recently been shown to be impaired in cells expressing mutated CSA and CSB proteins [22], hinting at the importance of ribosome biogenesis in the CS phenotype.

Although the localization of XPG within the nucleolus is well described [18], no study has established a clear role for XPG in RNAP1 transcription and more generally in ribosome biogenesis. The objectives of this work were to identify potential roles of the XPG protein in ribosomal DNA transcription, but also in rRNA maturation, by studying these processes in both wild type and XP-G/CS cell lines, hoping to open new therapeutic pathways for the combined disease of *Xeroderma Pigmentosum* and Cockayne Syndrome. We managed to demonstrate that a stable XPG protein is required for proper rDNA transcription, as well as rRNA maturation.

## Material & methods

### Cell culture

Wild type (Wt) and XPG-depleted (Xpg^*-/-*^) murine embryonic fibroblasts (MEFs) [23] were grown in Dulbecco’s Modified Eagle’s Medium (DMEM) (Sigma, D5796), supplemented with 1% penicillin/streptomycin (Gibco, 11548876) and 20% heat-inactivated fetal bovine serum (Corning, 35-015-CV). MRC5-SV, XPCS1RO-SV [24], GM14930-SV (Coriell, GM14930) and GM14931-SV (Coriell, GM14931) were grown in DMEM (Sigma, D5796), supplemented with 1% penicillin/streptomycin (Gibco, 11548876) and 10% non-heat-inactivated fetal bovine serum (Corning, 35-015-CV).). All these cell lines were grown at 37°C with 5% CO_2_ and 3% O_2_ to reduce oxidative damage. XPCS1RO-SV+XPG-GFP [25] were grown with 200 μg/mL of G418 (Sigma Aldrich, G8168-50ML). They were grown at 37°C with 5% CO_2_.

### RNA Fluorescence In Situ Hybridization (FISH)–Immunofluorescence

RNA FISH–Immunofluorescence was performed as in [3], on 18-mm coverslips with 3.5 μL of probe (10 ng/mL) in 70 μL of hybridization mix. The subsequent steps were performed as previously described. Sequences of the probes can be found in S1 Table. At least 3 biological replicates of the experiment were performed.

### Imaging & analysis

Images were taken on Andor Spinning Disk with a 40X oiled objective for MEFS and on Z1 Axioimager upright (Zeiss) with a 40X non-oiled objective before being analysed on ImageJ (NIH). For every condition of every experiment, at least 25 cells were quantified using a macro developed by P-O M. Nuclei as well as nucleoli were delimited using DAPI staining images: contrary to nuclei, nucleoli are not stained by DAPI, appearing as dark areas.

### Chromatin Immunoprecipitation (ChIP)

Cells were grown to 60–80% confluency before being washed with cold PBS and fixed with 1% formaldehyde diluted in PBS (Thermo Fisher, 28908) during 10 minutes. Fixation was quenched with 0.125 M glycine and cells were washed and collected in PBS+Protease Inhibitor Cocktail (PIC) 1X (cOmplete EDTA-Free Protease Inhibitor Cocktail, Roche, 5056489001) and pelleted by centrifugation 8 minutes at 4°C, 800 G. Cells were washed with Wash solution (PBS and 0,05% Active Motif detergent 37571, and PIC 1X) and PBS+PIC 1X and resuspended in Nuclear lysis buffer (10 mM Tris-HCl pH 8, 10 mM NaCl, 0,2% NP-40 and PIC 1X) before being dounced with a douncer B. After 10 minutes on ice, cells were resuspended in sonication buffer (50 mM Tris-HCl pH 8, 10 mM EDTA (Euromedex, EU0084) and 0,25% SDS (Euromedex, EU0360)) before sonication in Covaris S220 (Covaris, 500217; Peak Intensity Power 14 watts, Duty Factor 10% Cycler per burst 200, 9 minutes). Dosing of the chromatin was performed with Quantus Fluorometer (Promega, E6150) and chromatin was diluted in TE1X (10 mM Tris-HCl pH 8, 1 mM EDTA). Optimal Input quantity was stored at -20°C while optimal quantity for IP was incubated overnight at 4°C on wheel with Protein G magnetic beads (Thermo Fisher, Dynabeads Protein G 10004D) associated with either anti-mouse IgG (Diagenode, C15400001), anti-RPA194 (C-1) (Santa Cruz, sc-48385), or anti-UBF (F-9, X) (Santa Cruz, sc-13125 X) in DRIP Binding Buffer 1X (10X: 100 mM sodium phosphate, pH 7.14, 1.4 M NaCl and 0.5% Triton X-100). After IP, beads were washed twice with RIPA Low-salt buffer (TE 1X, 0.1% SDS, 140 mM NaCL, 0.1% Na-Deoxycholate and 1% Triton X-100) + PIC 1X, twice with RIPA High-salt buffer (TE 1X, 0.1% SDS, 500 mM NaCl, 0.1% Na-Deoxycholate and 1% Triton X-100) + PIC 1X, twice with LiCl buffer (TE 1X, 250 mM LiCl, 0.5% Na-Deoxycholate and 0.5% NP-40) + PIC 1X and twice in TE 1X + PIC 1X. DNA and proteins were then eluted in elution buffer (TE 1X, 1% SDS) on a thermomixer 1400 rpm at 37°C for 20 minutes, two times, before DNA was decrosslinked from proteins 5 hours at 65°C with 0.3 M NaCL and 5 μg of Proteinase K (Millipore, 124568) and 30 minutes at 37°C with 5 μg RNAse A (Thermo Fisher, EN0531). DNA was then extracted with QIAGEN MinElute PCR purification kit (Qiagen, 28004) following manufacturer’s instructions. DNA obtained from IP was diluted 10 times and DNA obtained from Input was diluted 100 times before proceeding to qPCR.

### DNA-RNA Immunoprecipitation (DRIP)

Cells were grown to 60–80% confluency before being washed with cold PBS and collected by trypsinization. DNA was extracted from cell pellets using DNEasy Blood and Tissue kit (QIAgen, 69504) following manufacturer’s instructions. Optimal quantity of DNA was cut with the following enzymes cocktail: BSA 1X, NEB Buffer 2 1X, 30 U *Bsr*GI (NEB, R0575S), 30 U *Eco*RI-Hf (NEB, R3101), 30 U *Ssp*I-Hf (NEB, R3132), 30 U *Hin*dIII (NEB, R0104S), 30 U *Xba*I (NEB, R0145S) and Spermidine 0.5X (Sigma-Aldrich, 05292-1ML-F) overnight at 37°C. Digested DNA was divided into two samples: one treated with 3.75 U RNase H (NEB, M0297L) for 5 hours at 37°C and then inactivated with 1 μL EDTA 0.5 M while the other was kept at 4°C. The optimal quantity of DNA for input was stored at -20°C and the optimal quantity of DNA for IP, treated and non-treated with RNase H and diluted in DRIP Binding Buffer 1X, was incubated on wheel overnight at 4°C with anti-S9.6 (Merck-Millipore, MABE1095) or without antibody. Magnetic beads coupled with protein G were added to the DNA mixes and incubated on wheel at 4°C for 4 hours before being washed as the ChIP samples. Decrosslinking was performed overnight at 65°C with 5 μg of Proteinase K (Millipore, 124568) and extraction was done with NucleoSpin Gel and PCR clean-up kit (Macherey-Nagel, 740609.50). DNA was diluted ten times before proceeding to qPCR. As the S9.6 antibody may cross-react with other structures, the signal obtained in RNAseH-treated samples was subtracted to the signal obtained in untreated samples to ensure that only R-loops signal was taken into account.

### qPCR

Cycle threshold was measured using Power SYBR Green PCR Master Mix (Applied Biosystems, 10209284) with CFX-Connect (Bio-Rad, 1855201). Obtained data were then normalized in Input Percentage or Fold Gene expression over housekeeping genes. At least 2 biological replicates were analysed for every experiment. Primers sequence are available in S2 Table.

### Total RNA extraction

Total RNA was extracted using RNAzol RT (Sigma-Aldrich, R4533) following manufacturer’s instructions. Briefly, cells 60–80% confluent were collected in 1 mL RNAzol RT (Sigma-Aldrich, R4533) and centrifugated to separate RNA from DNA & proteins. RNA was precipitated with 100% isopropanol and washed twice with 75% ethanol before being suspended in water without drying and dosed with Nanodrop. 3 independent biological replicates were extracted before being analysed by Northern Blot.

### Northern Blot

4 μg of RNA was separated on a 0.8% agarose gel with 1.2% formaldehyde in T/T buffer (30 mM Tricine (Santa Cruz, CAS 5704-04-1), 30 mM triethanolamine (Supelco, 8223415000)) before being transferred on a positively charged nylon membrane (Roche, 11-209-299-001) by downward capillary transfer in mildly alkaline condition. Membranes were prehybridized one hour at 42°C with ULTRAhyb-Oligo buffer (Life/ThermoFisher, AM8663) before being hybridized overnight at 42°C with 10 μM 5’ biotinylated probe in ULTRAhyb-Oligo buffer. They were washed twice with stringency wash buffer (2X SSC, 0.5% SDS from stock 20X SSC (Life/ThermoFisher, AM9770)) at 42°C. Afterwards, membranes were washed two times with PBS and 0.5% SDS, three times with blocking buffer (PBS, 0.5% SDS, 0.1% I-Block Reagent (Thermo Fisher, T2015)), once with conjugate solution (1 μL of Streptavidin-AP conjugate (Life/ThermoFisher, #S921) in 10 mL blocking buffer), once with blocking buffer, three times with washing buffer 1X, twice with assay buffer (0.1 M diethanolamine (Supelco, 1162051000), 1 mM MgCl_2_, pH 10.0) and covered in CDP-Star® Luminescent Substrate (0.25 mM Ready-To-Use) (Life/ThermoFisher, T2146) before being revealed by chemiluminescence in a Chemidoc Touch (Bio-Rad, 1708370). Membranes were stripped by washing twice in boiling NB strip buffer (0.1X SSC, 0.5% SDS) and cooling down, then restarting from prehybridization with another probe. The sequences of the oligonucleotide probes are available in S3 Table. Signals were quantified using the ImageJ software and normalized using Ratio of Multiple Precursors method such as described in [26]: briefly, the signal of a specific precursor is normalized by the Primary Transcript Plus (PTP), upper band formed by the co-migration of 47S, 46S and 45S rRNA, in control and mutated cells. The precursor/PTP ratio in mutated cells is then normalized by the precursor/PTP ratio in control cells. All the ratios are then submitted to log_2_ and combined in a single graph.

### Statistical analysis

All statistical tests were performed with GraphPad Prism version 8.1.0. ns: not significant; *: P<0.05; **: P<0.01; ***: P<0.001; ****: P<0.0001.

## Results

### 47S quantity is increased in the absence of XPG

**Fig 1:**
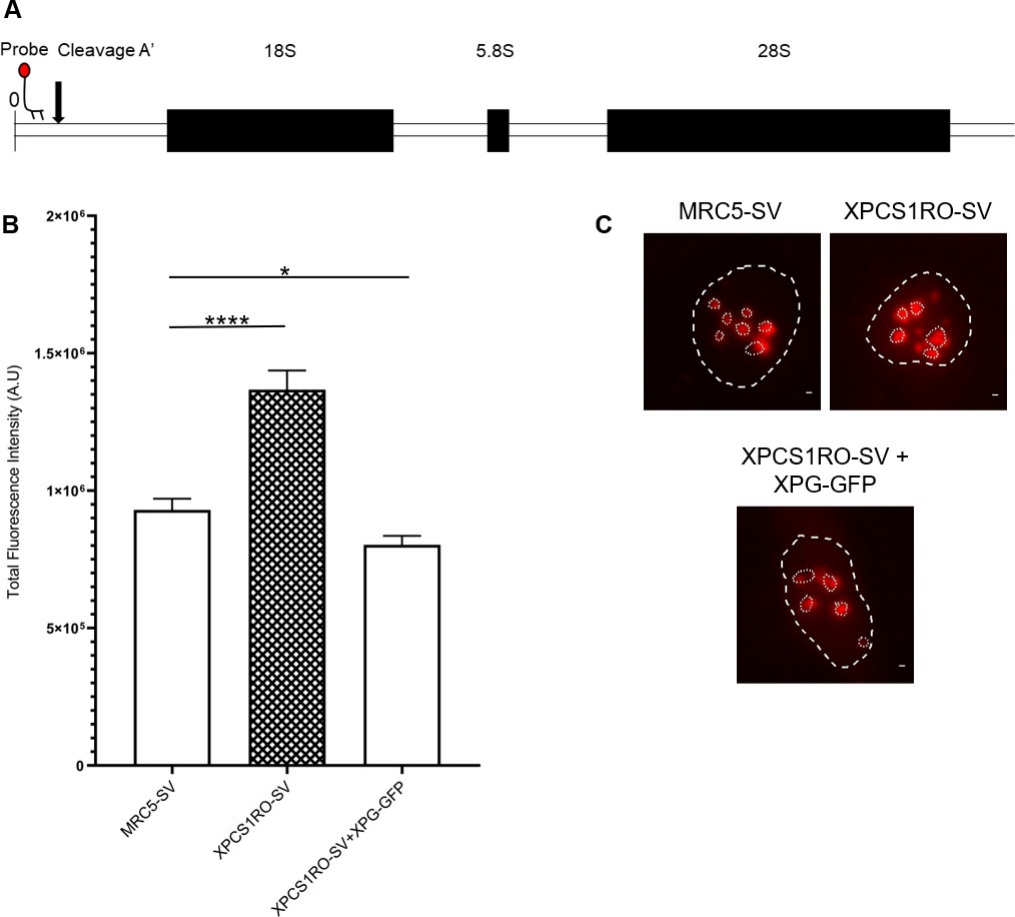
47S pre-rRNA quantity is increased in the absence of XPG. A) Schematic representation of rDNA unit and the 47S pre-rRNA probe localization. B) Quantification of the fluorescent signal in the nucleoli of MRC5-SV, XPCS1RO-SV and XPCS1RO-SV+XPG-GFP cell lines. C) Representative images of each cell line of B). Per panel 1 cell is depicted, and the nucleus and nucleoli borders are depicted. Scale bar: 10 μm. Error bars represent the SEM of three independent experiments. At least 25 nuclei were quantified for each experiment. Statistical significance was determined using unpaired t-tests in GraphPad Prism 8.1.0. *: p-value < 0.05; ****: p-value < 0.0001.

Because the XPG protein is located in the nucleolus and associated with CSB, TFIIH and RNAP1, we wondered if its absence could have an effect on the transcriptional activity of RNAP1. To assess this, we measured by RNA Fluorescence in Situ Hybridization (RNA FISH) the quantity of 47S pre-rRNA, the first product of RNAP1, which is cleaved rapidly and early during the maturation process [27]. For this, we used a specific oligonucleotide probe, directed against the 5’ of the 47S rRNA, upstream of cleavage site A’ (Fig 1A). Using this probe, the amount of newly transcribed 47S was quantified in MRC5-SV (wild type cell line), XPCS1RO-SV (immortalized cell line derived from a XP-G/CS patient with a XPG protein containing a frameshift mutation at aa 926 and a premature stop codon at aa 980 [28]), XPCS1RO-SV+XPG-GFP (rescue cell line) cell lines (S1 Fig).

Interestingly, instead of a reduction of the 47S signal, a higher level of 47S was measured in the nucleoli of XPCS1RO-SV cells. In the XPCS1RO-SV cell line complemented with XPG-GFP, the level of 47S rRNA decreased to a level slightly lower than in MRC5-SV cells (Fig 1B and 1C). This surprising result prompted us to measure the 47S amount in two other independent XP-G/CS cell lines: GM14931 (Coriell), presenting a premature stop codon at aa 659 [28] and GM14930 (Coriell), presenting one allele similar to GM14931 and the other allele coding for a XPG protein of only 262 aa [28] (S1 Fig).

In accordance with the results observed in XPCS1RO-SV cell lines, in both the GM cell lines, an increase of the level of 47S compared to the wild type cells was observed (S2B and S2C Fig). This result suggests that the absence of a stable XPG protein in human cells leads to an increased amount of 47S pre-rRNA.

To verify whether this feature was common to other mammalian XPG models, the RNA FISH experiment was performed in Mouse Embryonic Fibroblasts (MEFs) [23], either in wild-type (Wt) cells or XPG-depleted (Xpg^*-/-*^) cells. As in the human cell lines, the quantity of 47S rRNA was increased in the Xpg^*-/-*^ MEFs (S2C and S2D Fig), confirming that the absence of a stable XPG protein leads to an increased level of 47S in mammalian cells. The increase in 47S quantity in the nucleoli is related to the lack of a functional XPG protein, and is not cell-line specific.

### The absence of XPG changes the ribosomal DNA chromatin environment

Two possible hypotheses could explain this increase in 47S quantity: either an increase of the transcriptional activity of RNAP1, leading to the production of a greater quantity of pre-rRNA 47S, or a perturbation in 47S maturation, causing an accumulation of this primary product due to the inability of the cell to properly proceed to its maturation.

**Fig 2:**
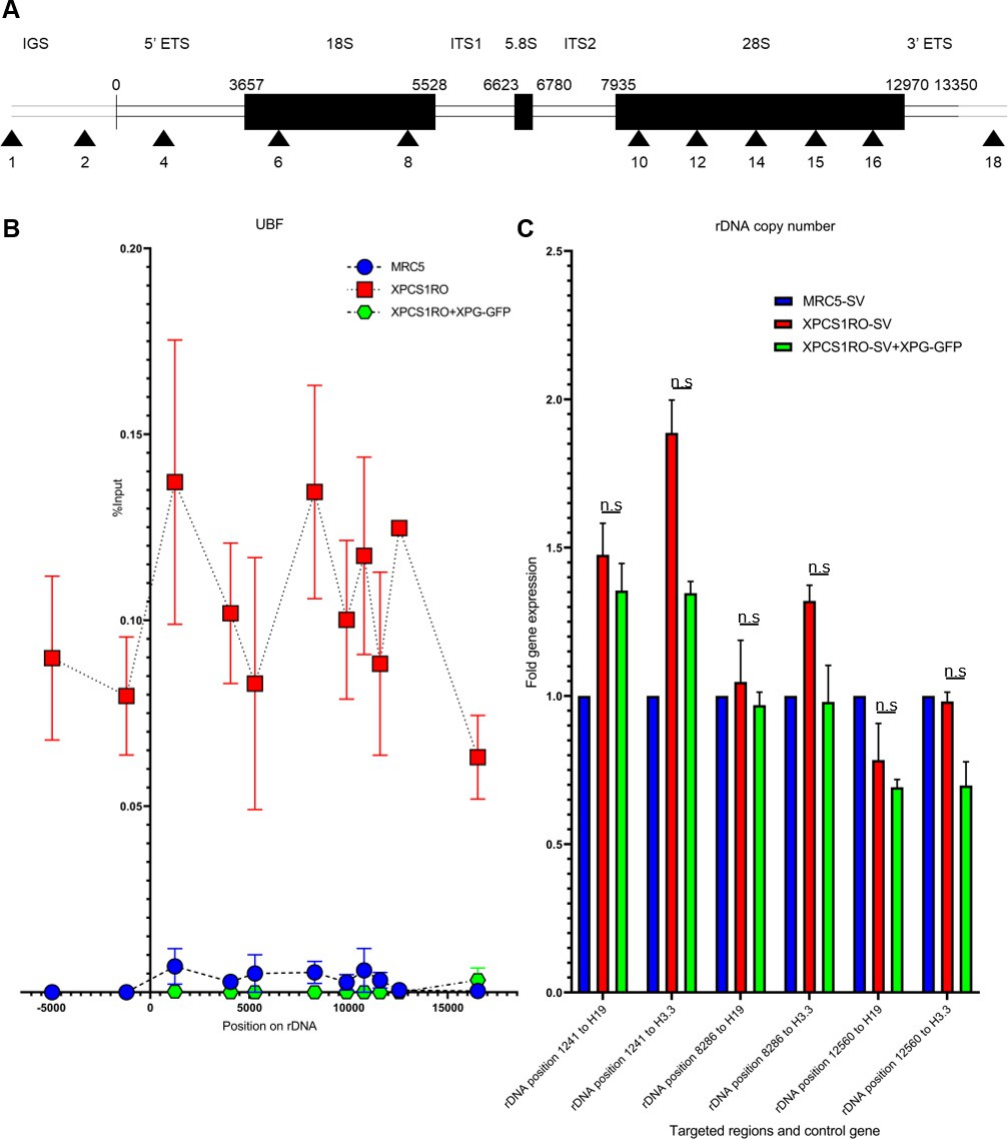
The absence of a stable XPG protein leads to an increased binding of UBF along rDNA unit. A) Scheme of the human rDNA unit with the position of all the primers used. IGS: InterGenic Spacer; ETS: External Transcribed Spacer; ITS: Internal Transcribed Spacer. B) ChIP-qPCR using UBF antibody in MRC5-SV, XPCS1RO-SV and XPCS1RO-SV+XPG-GFP cell lines. Signal was quantified as percentage of input DNA. Error bars represent the SEM of two independent experiments. C) Fold gene expression of three rDNA regions, relatively to two housekeeping genes, in MRC5-SV, XPCS1RO-SV and XPCS1RO-SV+XPG-GFP cell lines. The error bars represent the SEM of 3 biological replicates. Statistical significance was determined using the Holm-Sidak method in GraphPad Prism 8.1.0, with ⍺ = 0.05. n.s: Not significant.

To investigate rDNA transcription in the absence of a stable XPG protein, chromatin immunoprecipitation coupled with quantitative PCR (ChIP-qPCR) with an antibody targeting Upstream Binding Factor (UBF) [29] was performed in MRC5-SV, XPCS1RO-SV and XPCS1RO-SV+XPG-GFP. 11 primers along the sequence of the ribosomal genes and in non-transcribed flanking regions were used to establish a map of the binding of this protein along the rDNA (Fig 2A).

In the absence of a functional XPG protein, UBF binding to the rDNA is increased compared to the control cells. In the rescue cell line XPCS1RO-SV+XPG-GFP, UBF binding along the rDNA is similar to the profile established in WT MRC5 cell line. This result shows that the binding of UBF along the rDNA is modified in the absence of a stable XPG protein (Fig 2B). As UBF reflects the number of active copies of rDNA, this result could mean that the number of active copies of rDNA is increased in the absence of XPG. However, the number of rDNA copies varies between individuals [30]. To verify that the increased number of copies is related solely to the increased binding of UBF, the number of basal rDNA copies was estimated by qPCR in the MRC5-SV, XPCS1RO-SV and XPCS1RO-SV+XPG-GFP cell lines. No difference of the number of copies was observed between XPCS1RO-SV and XPCS1RO-SV+XPG-GFP cell lines, implying that the augmentation of the number of active copies of rDNA is solely related to the absence of a functional XPG protein (Fig 2C). Intriguingly, when ChIP-qPCR using an antibody targeting RNA Polymerase 1 (RNAP1) was performed in these three cell lines, a decreased RNA Polymerase 1 binding along the rDNA was measured in the XPCS1RO-SV cell line, a pattern that the rescue of the cell line with a wild-type XPG protein failed to rescue (S3 Fig). However, similar results of increased UBF binding without affecting RNAP1 binding were previously observed [21]. This result suggests that the lack of a stable XPG protein leads to an increased number of active copies of rDNA.

### Unresolved R-loops are increased along rDNA sequence in absence of XPG

**Fig 3:**
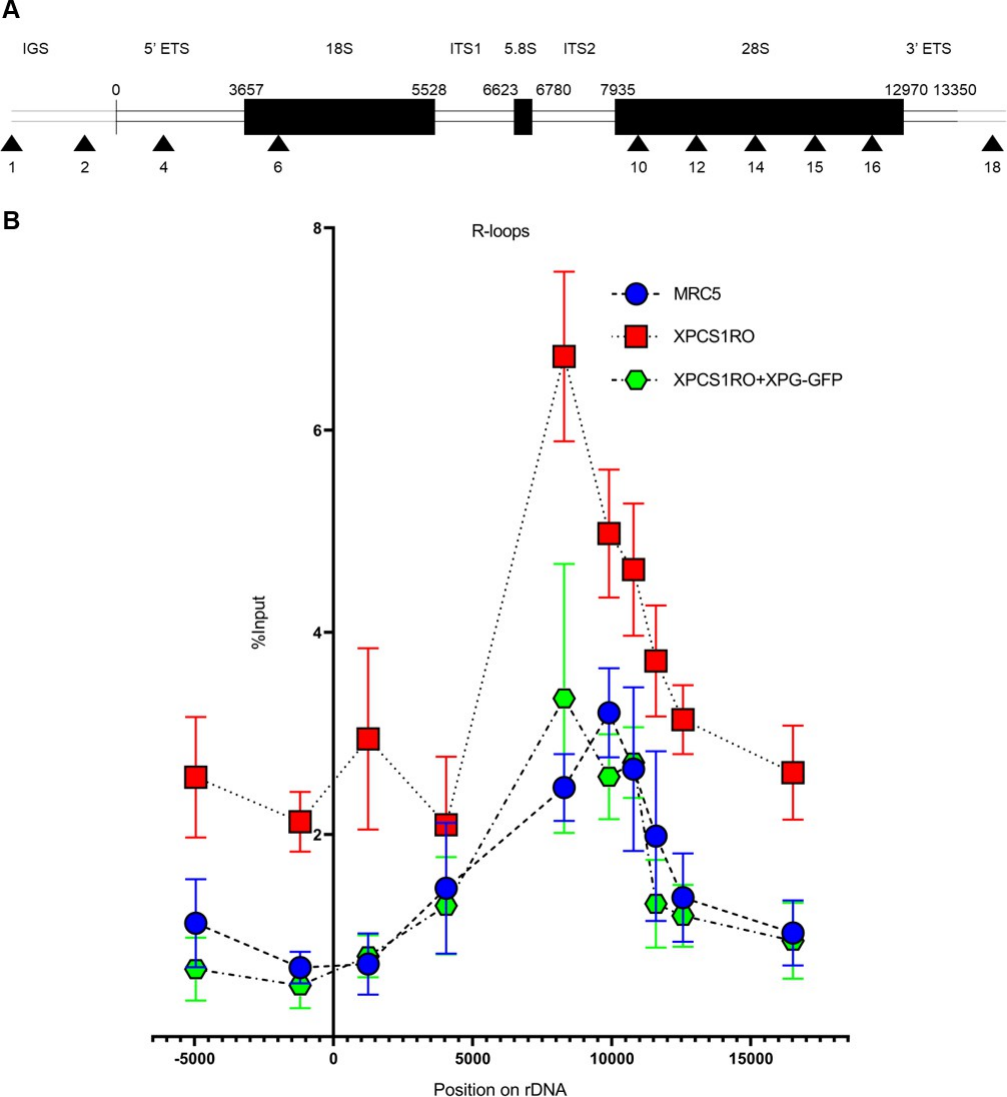
The unresolved R-loops are increased along the rDNA unit in the absence of XPG. A) Scheme of the human rDNA unit with the position of all the primers used. IGS: InterGenic Spacer; ETS: External Transcribed Spacer; ITS: Internal Transcribed Spacer. B) DRIP-qPCR in MRC5-SV, XPCS1RO-SV and XPCS1RO-SV+XPG-GFP cell lines. Signal was quantified as percentage of input DNA and normalized using controls treated with RNase H. Error bars represent the SEM of three independent experiments.

In actively transcribed regions, nascent RNA is emerging upstream of the moving RNA polymerase. This nascent RNA is naked, and can form hybrid structures in complex with the double-stranded DNA. These hybrid structures are called R-loops, reflecting their similarity with D-loops [31]. R-loops tend to form in transcriptionally active regions and/or GC-rich regions, such as ribosomal genes. Recently, it has been shown that the XPG protein is in charge of processing some of the R-loops formed upon Double Strand Breaks induction, to start a repair process known as Transcription-Associated Homologous Recombination Repair (TA-HRR) [16]. In a similar fashion, XPG could be involved in the processing of R-loops produced along the rDNA, while RNAP1 is actively transcribing. To verify this hypothesis, DNA/RNA ImmunoPrecipitation (DRIP) experiment in association with qPCR were performed. Briefly, by using an antibody targeting R-loops following DNA digestion by an enzymatic cocktail, and proceeding as for the ChIP experiments, the number of unresolved R-loops along the ribosomal genes was established (Fig 3A).

In the XPCS1RO-SV cell line, a higher number of R-loops precipitated along the rDNA was measured compared to the wild-type cell line, particularly in the 5’ region of the 28S rRNA, a known hotspot for R-loops formation [32]. To verify that this result depends on the absence of a stable XPG protein, DRIP-qPCR experiment was performed in the XPCS1RO-SV cell line corrected with XPG-GFP. In this cell line, the R-loops binding profile along the rDNA was very similar to the one established in the wild type cell line (Figs 3B and S4). This result prompted us to think that a functional XPG protein contributes to reduce the number of R-loops along the rDNA.

### The maturation of rRNA is impaired in the absence of a functional XPG protein

**Fig 4:**
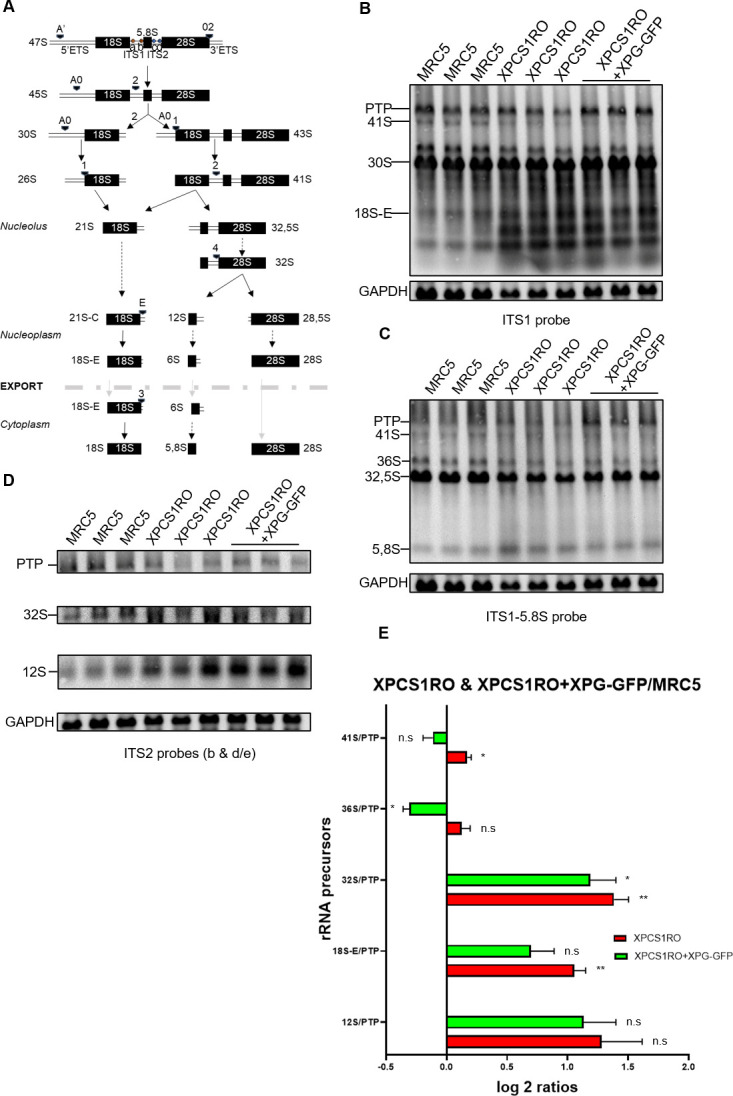
The absence of XPG leads to an increase of alternative rRNA precursors. A) Scheme of the processing of the rRNA in human cells with the location of the Northern Blot probes. a: Probe ITS1; b: Probe ITS1-5.8S; c: Probe ITS2b; d: Probe ITS2 d/e; Arrows: Endonucleolytic cleavage; Dotted arrows: Action of an exoRNase; Grey arrows: Export through the nuclear membrane; Italic name: Localization of the precursor; Bold: Export of the precursor; Thick grey dotted line: Nuclear membrane. B-D) Northern blot analysis of the RNA of three biological replicates of MRC5-SV, XPCS1RO-SV and XPCS1RO-SV+XPG-GFP cell lines with the ITS1 (B), ITS1-5.8S (C) and ITS2b and d/e (D) probes. PTP: Primary Transcript Plus. E) Ratio Analysis of Multiple Precursors (RAMP) of various pre-rRNAs in XPCS1RO-SV and XPCS1RO-SV+XPG-GFP normalized to the same ratios in the MRC5-SV. The error bars represent the SEM of 3 biological replicates. Statistical significance was determined using the Holm-Sidak method relative to MRC5 value (0) in GraphPad Prism 8.1.0, with ⍺ = 0.05. n.s: Not significant; *: p<0.05; **: p<0.01.

Following transcription, 47S ribosomal RNA is cleaved several times, ultimately leading to three mature forms: 18S, 5.8S and 28S. We hypothesized that the increase of rRNA 47S observed in the absence of a functional XPG could be a consequence of an improper maturation process. To check the validity of this hypothesis, Northern Blot experiments with oligonucleotides probes hybridizing within the Internal Transcribed Spacers were performed, allowing the visualization of several non-mature precursors of the rRNA (Fig 4).

To precisely quantify the maturation of the rRNA in these three cell lines, the Ratio Analysis of Multiple Precursors (RAMP) method was applied. Briefly, the signal of a specific precursor is normalized on the Primary Transcript Plus (PTP) signal, upper band formed by the co-migration of 47S, 46S and 45S rRNA. The precursor/PTP ratio in mutated cells, XPCS1RO-SV and XPCS1RO-SV+XPG-GFP is then normalized by the precursor/PTP ratio in control cells, MRC5 cells. All the ratios are then submitted to log_2_. RAMP allows to check maturation *per se* by correcting discrepancies in initial RNA quantity and eventual losses during transfer. The resulting value for every precursor of interest has been regrouped in one graph (Fig 4E). A positive value indicates an increase of the quantity of this precursor in the cell line compared to the quantity of this precursor in the MRC5 cell line, while a negative value indicates a decrease of the quantity of this precursor in the cell line compared to the MRC5 cell line.

In XP-G/CS context, a slight increase of the rRNA precursor 41S was measured. When the XPCS1RO-SV cell line is rescued with a functional XPG protein, the 41S rRNA precursor signal is comparable to the signal observed in wild type cell line. The absence of a functional XPG protein increases the quantity of 41S rRNA precursor. Furthermore, in the XPCS1RO-SV+XPG-GFP cell line, we measured a decrease of the quantity of 36S rRNA precursor. Both 41S and 36S rRNA precursors come from alternative cleavage pathways: thus, the absence of a stable XPG protein could guide the cell towards alternative ways of maturation, and restoring a functional protein may promote the classical pathways of maturation.

An increase of the quantity of 32S rRNA precursor was measured in the XPCS1RO-SV cell line. In the rescue cell line, the quantity of this precursor is slightly decreased. We can hypothesize that the XPG protein is involved in the cleavage at site 4. Furthermore, a higher tendency of the rRNA precursor 12S was observed, and an increase of both 32S and 12S precursors has been previously described [33]. Finally, an increase of the precursor rRNA 18S-E was observed in the XPCS1RO-SV cell line. In the XPCS1RO-SV+XPG-GFP cell line, the signal of this precursor decreases to control values. A functional XPG protein may be required for the export or exonucleolytic trimming of this precursor. Overall, in the absence of a functional XPG protein, rRNA maturation is impaired. Restoring a functional XPG protein, however, restores all the correct maturation steps except for the cleavage at site 4, which is only partially restored.

## Discussion

Here we show that the XPG protein functions in ribosome biogenesis: in the absence of a stable XPG protein, an increase of the pre-rRNA 47S quantity is measured. This may emerge from an increase of the number of active copies of rDNA, as well as from an impairment of rRNA maturation. Our results also indicate that a stable XPG protein is required to resolve the R-loops along the rDNA. These new roles for the XPG protein may contribute to explain the phenotypic diversity observed in XP-G/CS patients.

The absence of a functional XPG protein causes an increase of the 47S pre-rRNA in mammalian cells. This increase may be due to an augmentation of the number of active copies of rDNA: indeed, UBF binding to rDNAs reflects the number of active copies of rDNA [34] (Fig 2C). UBF is a member of the sequence-nonspecific class of high mobility group (HMG) proteins and function exclusively in RNAP1 transcriptional activity [35]. It allows the creation of the enhanceosome, a nucleosome-like structure, thus modifying rRNA transcription rate. As XPG is able to bend the DNA [36], its absence may hinder the transcription of the rDNA. To compensate, the cell must increase the number of active copies. As other regulation processes may be deregulated, the cell overcompensates, thus producing more 47S. Interestingly, an increased binding of UBF to rDNA has previously been linked with neurodegeneration in early childhood [21]. However, RNAP1 binding was not affected by the increased UBF binding, a pattern that has also been observed in our results. In the context of XP-G/CS, the increased UBF binding may explain the CS symptoms observed in patients expressing a truncated form of XPG protein.

An increase of the unresolved R-loops has been observed along the rDNA in the XPCS1RO-SV cell lines. These structures, formed by one strand of RNA and two strands of DNA, are frequent in transcribed regions. As such, it is not surprising to find them on the rDNA. XPG has been associated with R-loops processing in several cases [16, 37]. Our data shows that a stable XPG protein is required for the resolution of these structures in the nucleoli. The accumulation of R-loops has been extensively linked with neurodegenerative diseases. For example, trinucleotide repeat-associated diseases such as Huntington’s disease (CAG repeats), fragile X syndrome type E or A (CGG and CCG, respectively) or Friedreich Ataxia (GAA) have C/G-rich regions that are prone to R-loop formations, sometimes even double R-loops configuration [38]. Furthermore, the non-resolution of R-loops has been previously shown to impair RNAP1 transcriptional activity [39] which may also explain the activation of more rDNA copies; overall, R-loops accumulation, in addition with transcriptional deregulation, may contribute to the neurodegenerative phenotypes of XP-G/CS patients.

We also hypothesized that a defect of 47S rRNA maturation could be one of the reasons behind its accumulation. By using the RAMP method, we were able to analyze maturation *per se*: the double normalization, first over the PTP and then between cell lines ensure that discrepancies in initial RNA quantity and biological variations are corrected (GAPDH band in Fig 4B–4D). This permits a more precise study of pre-rRNA processing than by directly comparing band intensities [26]. An increase of the quantity of the 41S rRNA precursor in the absence of a functional XPG protein has been observed. This precursor is formed when cleavage at site 1 happens prior to cleavage at site 2 [40]. This is not the major pathway of rRNA maturation, and the absence of a stable XPG protein could force the cell to go through alternative ways of maturation. Furthermore, when the XPCS1RO-SV cells are complemented with a functional XPG protein, a decrease of the 36S rRNA precursor, another product of alternative cleavages, was measured, strengthening our hypothesis. XPG could be directly involved in the cleavages during the maturation of rRNA, as it is a member of the FEN-1 superfamily, which has previously been shown to cleave RNA, or it could play a structural role in these steps. An increase of the quantity of the 32S rRNA precursor has also been observed. In the complemented cell line, this increase was lowered. When cleaved at site 4, the 32S rRNA forms two precursors: the 12S and the 28.5S rRNA. As evocated earlier, XPG could be directly involved in the cleavage at site 4. However, the XRN2 protein has also been shown to lead to an increase in the quantity of 32S rRNA when depleted [33], and, interestingly, an increase of the 12S precursor, which has a higher tendency in our results as evocated earlier. Furthermore, XRN2 trims the 36S rRNA precursor of its nucleotides in 5’ [40], and 36S precursor is decreased when we restore a functional XPG protein. Thus, XPG and XRN2 may be associated in the process of rRNA maturation: in the absence of a stable XPG protein, XRN2 cannot properly participates in the maturation of 32S and 12S rRNA. However, when restoring XPG protein, XRN2 performs better in the trimming of the 36S precursor and slightly better in the maturation processes of the 32S precursor.

The increase of the 18S-E rRNA precursor in the XPCS1RO-SV cell line is also remarkable, as it has been recently observed in cell lines with mutated CSA and CSB protein [22]. This precursor is cleaved at site 3 to form the mature 18S. This endonucleolytic cleavage performed by NOB1 happens in the cytoplasm [41, 42]. Before being exporter, 18S-E must be trimmed of a few nucleotides by the poly-A specific ribonuclease (PARN) [43]. As XPG protein has only been located in the nucleus thus far, it may either be necessary for PARN activity or acts in collaboration with it to directly trim the 18S-E precursor. As the 18S-E is a GC-rich substrate compared to the ones usually processed by PARN, XPG may facilitate PARN action by “pre-cleaving” the 18S-E. Restoring a functional XPG protein restores the quantity of 18S-E precursor to the control value, indicating that a functional XPG protein is indeed required for the processing of the 18S-E rRNA precursor. Overall, a functional XPG protein seems necessary to perform correct rRNA maturation. When rRNA maturation is impaired, cell homeostasis may be modified in three manners: first, the quantity of mature ribosomes may be diminished, and it could affect the global level of translation. Secondly, it has been shown that the ribosome pool is very heterogeneous, with for example subsets of specialized ribosomes set to act at precise timepoints of the development or in specific tissues [44], and a change in pre-rRNA processing could impair the actions of these specialized ribosomes. Finally, even though aberrant ribosomes are submitted to quality-control mechanisms [45], some precursors rRNA have been shown to be able to participate in translation in *Saccharomyces cerevisiae* [46, 47], favouring the translation of certain types of mRNA over others [48]. Overall, the impairment of ribosome biogenesis may contribute to explain the severe phenotypes associated with XP-G/CS.

## Conclusion

We demonstrated that the presence of a truncated XPG protein modifies the homeostasis of the cell at several levels: more rDNA copies are activated, possibly to overcompensate the loss of transcriptional activity caused by the lack of a properly functioning XPG protein and an accumulation of R-loops. Furthermore, rRNA maturation is impaired in the absence of a stable XPG protein, with alternative pathways of maturation being followed and abnormal accumulations of certain precursors. These defects may contribute to explain the neurodegenerative phenotypes observed in XP-G/CS patients and open new therapeutic leads for this deadly disease.

## Supporting information

S1 Table Sequences (5’ to 3’) of the probes used in RNA FISH analysis. (TIF)

S2 Table Sequences (5’ to 3’) of the qPCR primers used for ChIP, DRIP, and qPCR analysis of the ribosomal DNA. Position is the middle of the PCR product, related to the transcription start site (TSS).
(TIF)

S3 Table Sequences (5’ to 3’) of the probes used for Northern Blot analysis. All probes are biotinylated in 5’.
(TIF)

S1 Fig Structure of the XPG protein in the cell lines used in this study. Cartoon depicting the structure of the XPG protein in MRC5-SV, XPCS1RO-SV, XPCS1RO-SV+XPG-GFP, GM14931-SV and GM14930-SV cell lines. UBM: Ubiquitin Binding Motif; PIP: PCNA Interacting Peptide.
(TIF)

S2 Fig 47S rRNA quantity is increased in *Xeroderma Pigmentosum*/Cockayne Syndrome cell lines and in XPG-depleted mice cells. A) Schematic representation of rDNA unit and the 47S pre-rRNA probe localization. B) Quantification of the fluorescent signal in the nucleoli of MRC5-SV, GM14931-SV and GM14930-SV cell lines. C) Representative images of each cell line of B). Per panel 1 cell is depicted, and the nucleus and nucleoli borders are depicted. Scale bar: 10 μm. D) Quantification of the fluorescent signal in the nucleoli of wild-type (Wt) and Xpg^*-/-*^ murine embryonic fibroblasts (MEFs). E) Representative images of each cell line of D). Scale bar: 10 μm. Error bars represent the SEM of three independent experiments. At least 25 nuclei were quantified for each experiment. Statistical significance was determined using unpaired t-tests in GraphPad Prism 8.1.0. **: p-value < 0.01; ****: p-value < 0.0001.
(TIF)

S3 Fig The binding of RNA Polymerase 1 along rDNA unit is not related to the XPG protein. A) Scheme of the human rDNA unit with the position of all the primers used. IGS: InterGenic Spacer; ETS: External Transcribed Spacer; ITS: Internal Transcribed Spacer. B) ChIP-qPCR using RNA Polymerase 1 antibody in MRC5-SV, XPCS1RO-SV and XPCS1RO-SV+XPG-GFP cell lines. Signal was quantified as percentage of input DNA. Error bars represent the SEM of two independent experiments.
(TIF)

S4 Fig S9.6 antibody signal along rDNA with and without treatment with RNaseH. A) Scheme of the human rDNA unit with the position of all the primers used. IGS: InterGenic Spacer; ETS: External Transcribed Spacer; ITS: Internal Transcribed Spacer. B) S9.6 antibody signal in MRC5-SV, XPCS1RO-SV and XPCS1RO-SV+XPG-GFP cell lines, treated and not treated with RNaseH. Signal with RNaseH was subtracted to signal without RNaseH to calculate the R-loops signal along rDNA unit. Error bars represent the SEM of three independent experiments.
(TIF)

S1 Raw images (PDF)
