# Supplementary figures and images for: A stable XPG protein is required for proper ribosome biogenesis: Insights on the phenotype of combinate Xeroderma Pigmentosum/Cockayne Syndrome patients
Source: PLoS One. 2022 Jul 8;17(7):e0271246. doi: 10.1371/journal.pone.0271246 (PMC9269744; doi:10.1371/journal.pone.0271246)

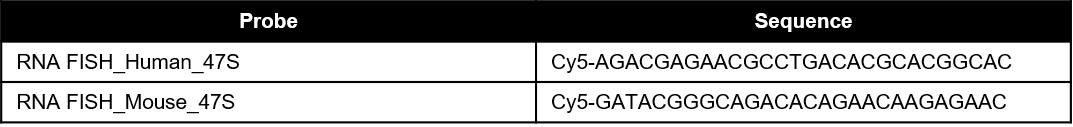

Supplement: S1 Table — (TIF) [file pone.0271246.s001.tif]

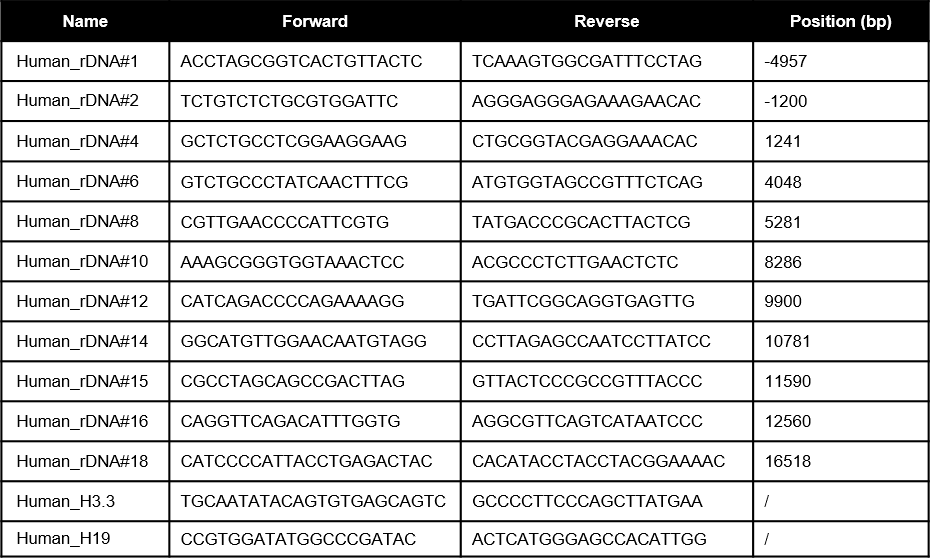

Supplement: S2 Table — Position is the middle of the PCR product, related to the transcription start site (TSS). (TIF) [file pone.0271246.s002.tif]

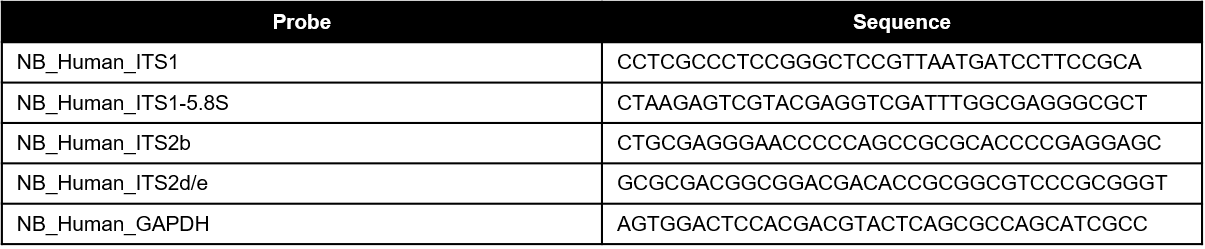

Supplement: S3 Table — All probes are biotinylated in 5’. (TIF) [file pone.0271246.s003.tif]

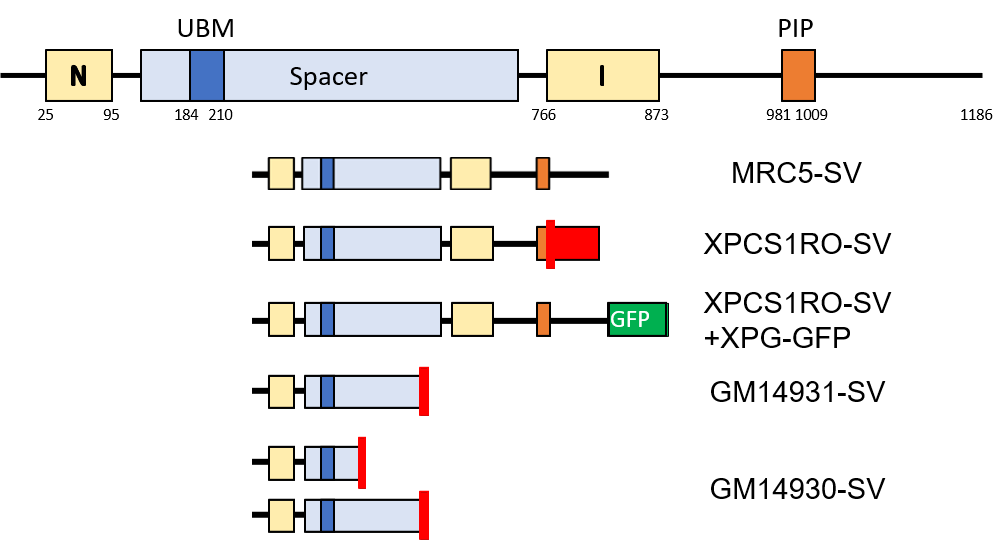

Supplement: S1 Fig — Cartoon depicting the structure of the XPG protein in MRC5-SV, XPCS1RO-SV, XPCS1RO-SV+XPG-GFP, GM14931-SV and GM14930-SV cell lines. UBM: Ubiquitin Binding Motif; PIP: PCNA Interacting Peptide. (TIF) [file pone.0271246.s004.tif]

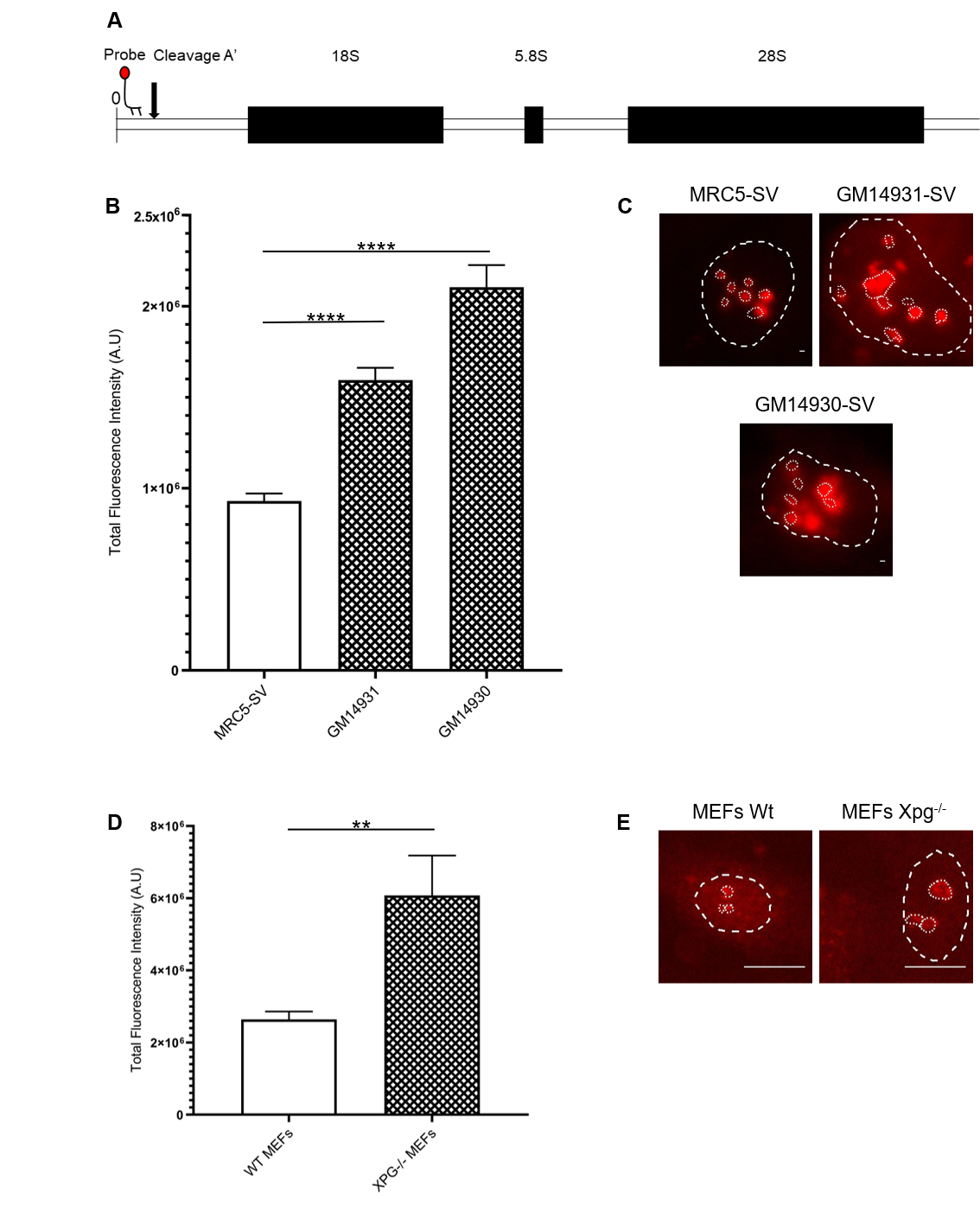

Supplement: S2 Fig — A) Schematic representation of rDNA unit and the 47S pre-rRNA probe localization. B) Quantification of the fluorescent signal in the nucleoli of MRC5-SV, GM14931-SV and GM14930-SV cell lines. C) Representative images of each cell line of B). Per panel 1 cell is depicted, and the nucleus and nucleoli borders are depicted. Scale bar: 10 μm. D) Quantification of the fluorescent signal in the nucleoli of wild-type (Wt) and Xpg-/- murine embryonic fibroblasts (MEFs). E) Representative images of each cell line of D). Scale bar: 10 μm. Error bars represent the SEM of three independent experiments. At least 25 nuclei were quantified for each experiment. Statistical significance was determined using unpaired t-tests in GraphPad Prism 8.1.0. **: p-value < 0.01; ****: p-value < 0.0001. (TIF) [file pone.0271246.s005.tif]

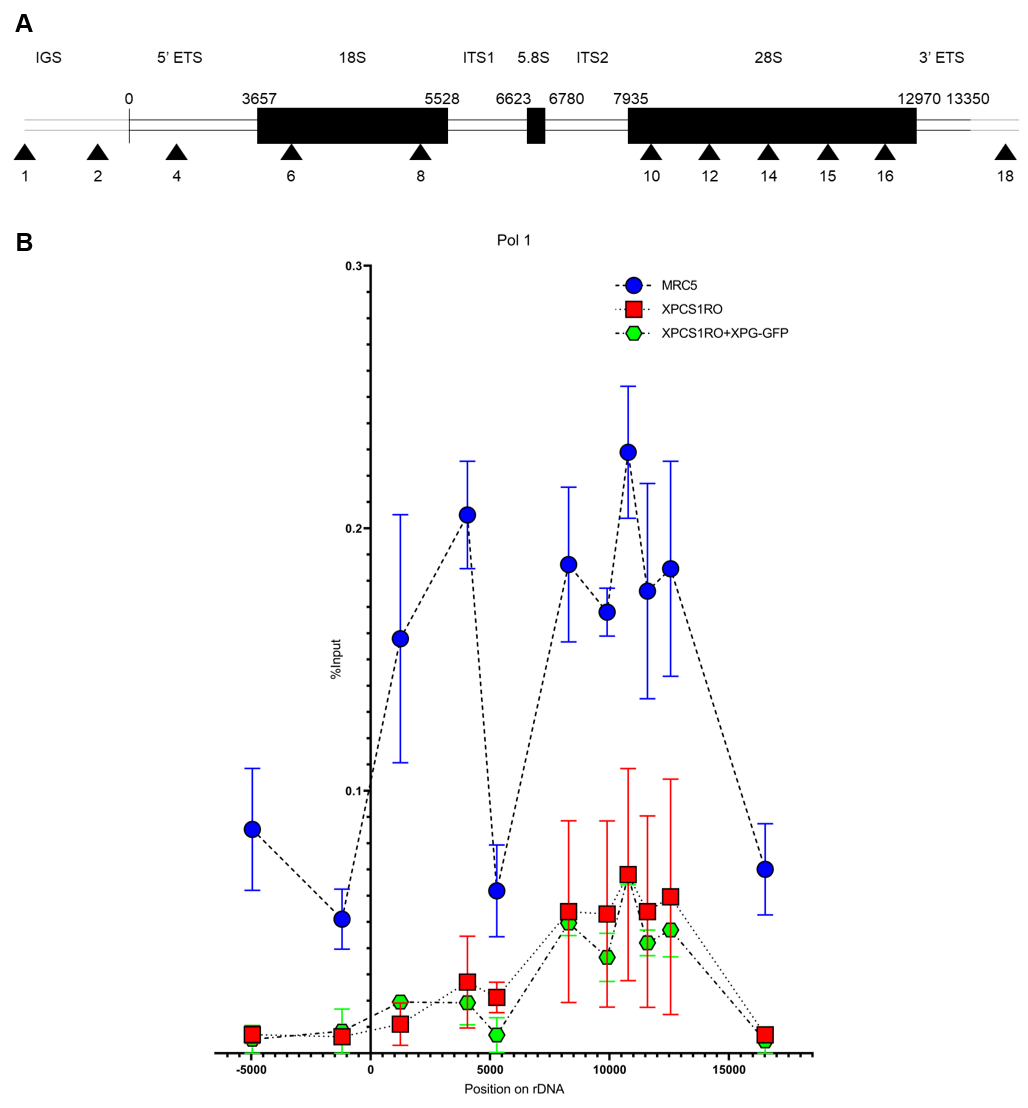

Supplement: S3 Fig — A) Scheme of the human rDNA unit with the position of all the primers used. IGS: InterGenic Spacer; ETS: External Transcribed Spacer; ITS: Internal Transcribed Spacer. B) ChIP-qPCR using RNA Polymerase 1 antibody in MRC5-SV, XPCS1RO-SV and XPCS1RO-SV+XPG-GFP cell lines. Signal was quantified as percentage of input DNA. Error bars represent the SEM of two independent experiments. (TIF) [file pone.0271246.s006.tif]

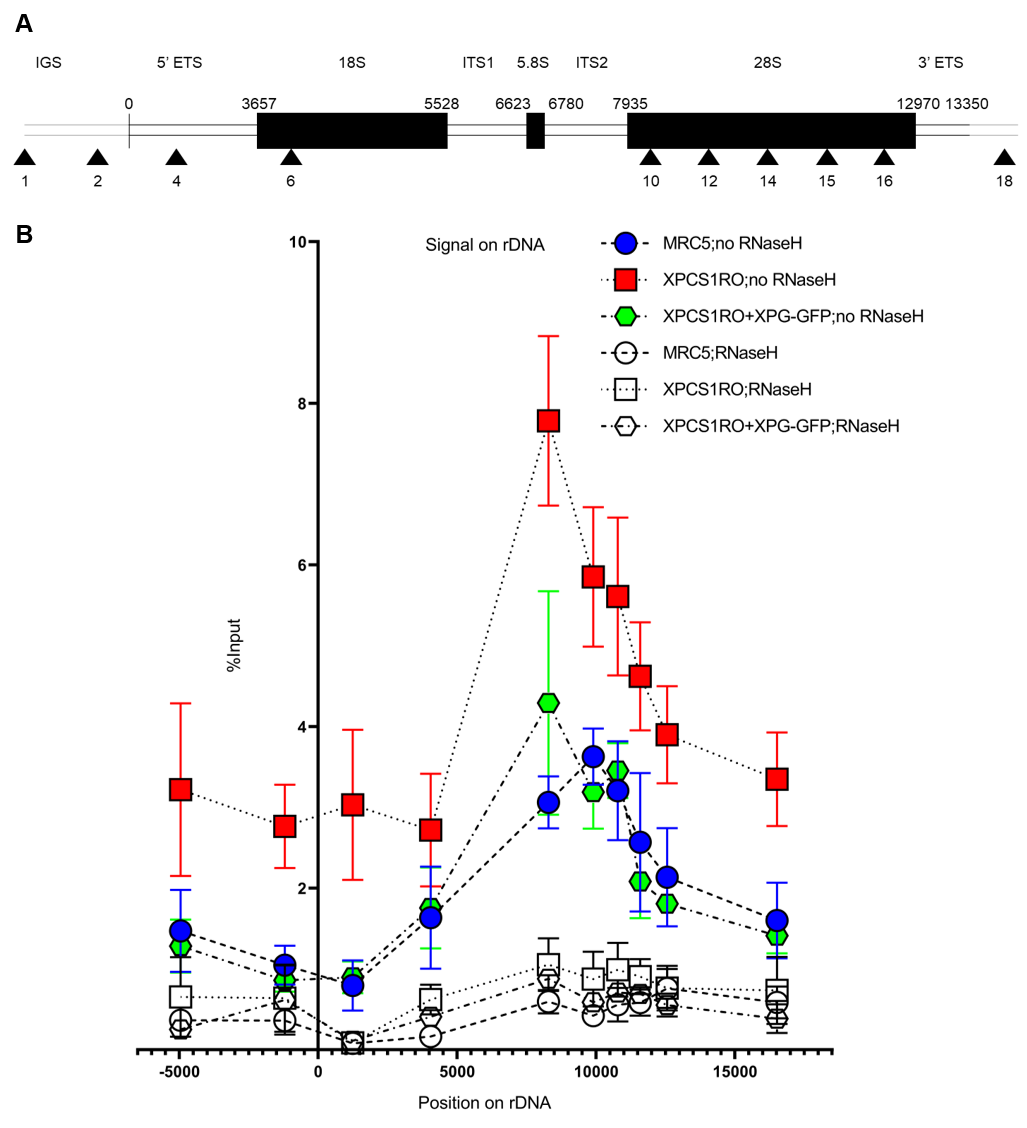

Supplement: S4 Fig — A) Scheme of the human rDNA unit with the position of all the primers used. IGS: InterGenic Spacer; ETS: External Transcribed Spacer; ITS: Internal Transcribed Spacer. B) S9.6 antibody signal in MRC5-SV, XPCS1RO-SV and XPCS1RO-SV+XPG-GFP cell lines, treated and not treated with RNaseH. Signal with RNaseH was subtracted to signal without RNaseH to calculate the R-loops signal along rDNA unit. Error bars represent the SEM of three independent experiments. (TIF) [file pone.0271246.s007.tif]
