# Supplementary material for: A stable XPG protein is required for proper ribosome biogenesis: Insights on the phenotype of combinate Xeroderma Pigmentosum/Cockayne Syndrome patients
Source: PLoS One. 2022 Jul 8;17(7):e0271246. doi: 10.1371/journal.pone.0271246 (PMC9269744; doi:10.1371/journal.pone.0271246)
Supplement: S1 Raw images — (PDF) [file pone.0271246.s008.pdf]

Agarose gel was revealed using Gel Doc XR (Bio-Rad, 170-8170).

Following transfer to a positively charged Nylon membrane (Roche, 11-209-299-001) RNAs of interest were probed with 5' biotinylated probes and revealed via chemiluminescence using Streptavidin-AP conjugate (Life/ThermoFisher, #S921) in Chemidoc Touch (Bio-Rad, 1708370), in Signal Accumulation Mode. Picture before saturation of a band of interest was used to quantify signal using the ImageJ software.

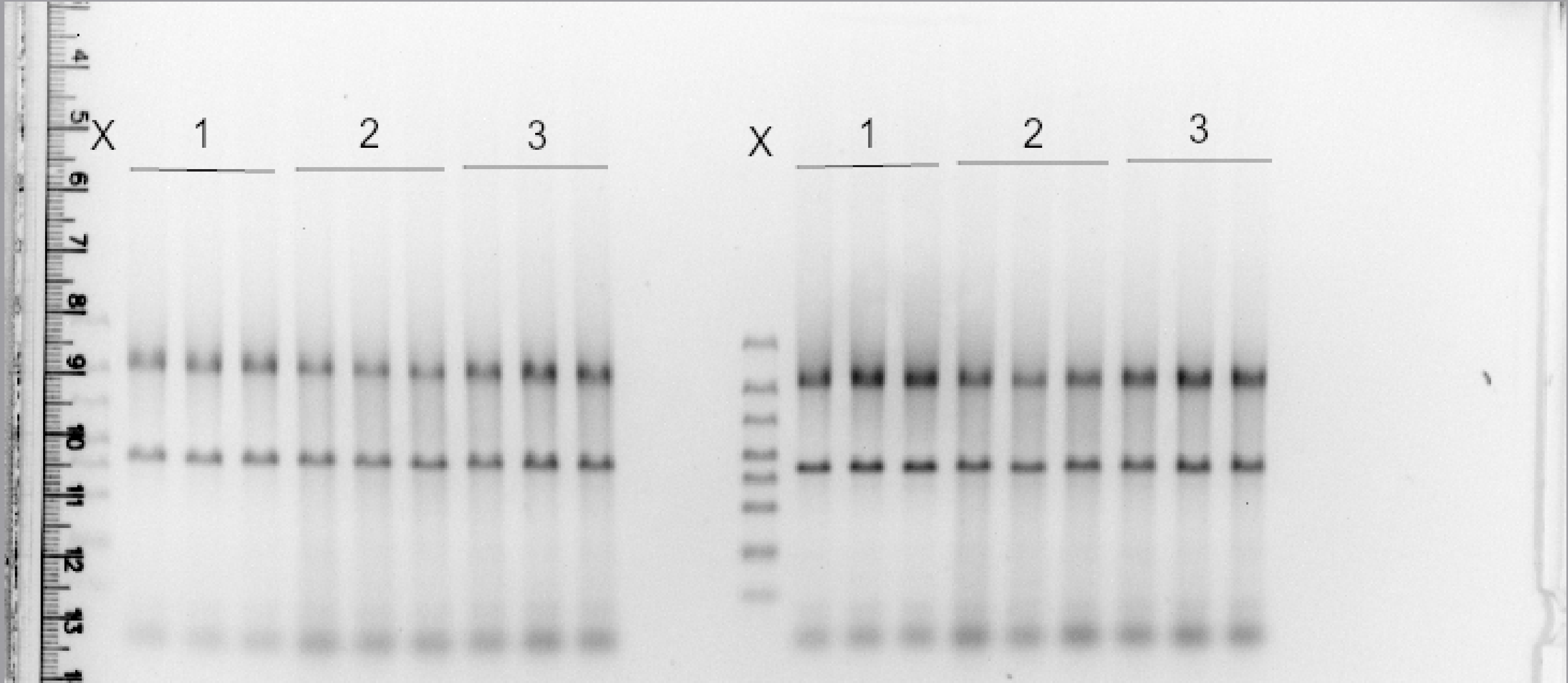

Original Northern Blot agarose gel

1 : 3 biological samples of MRC5-SV RNA
2 : 3 biological samples of XPCS1RO-SV RNA
3 : 3 biological samples of XPCS1RO+XPG-GFP-SV RNA
X : RiboRuler High Range RNA Ladder (Thermo Fisher, SM1821) ; does not appear on blot

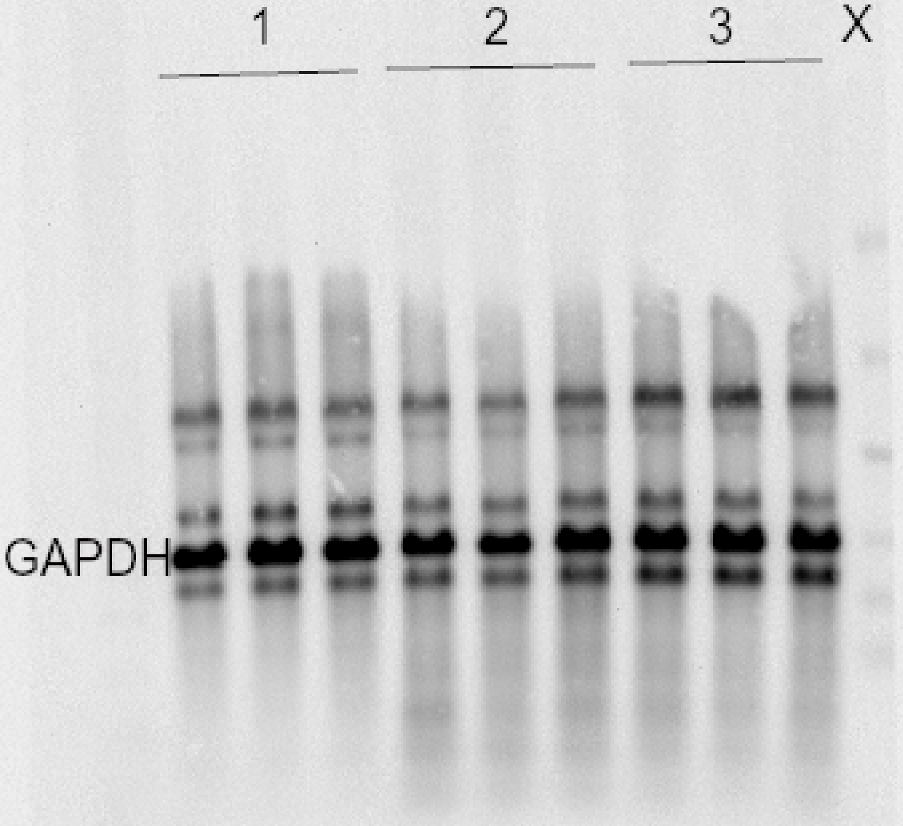

1 : 3 biological samples of MRC5-SV
2 : 3 biological samples of XPCS1RO-SV RNA
3 : 3 biological samples of XPCS1RO+XPG-GFP-SV RNA
X : DynaMarker Prestain Marker for RNA High (Biodynamics, DM260), not displayed because size is improper on T/T gel

GAPDH band in Figure 4D

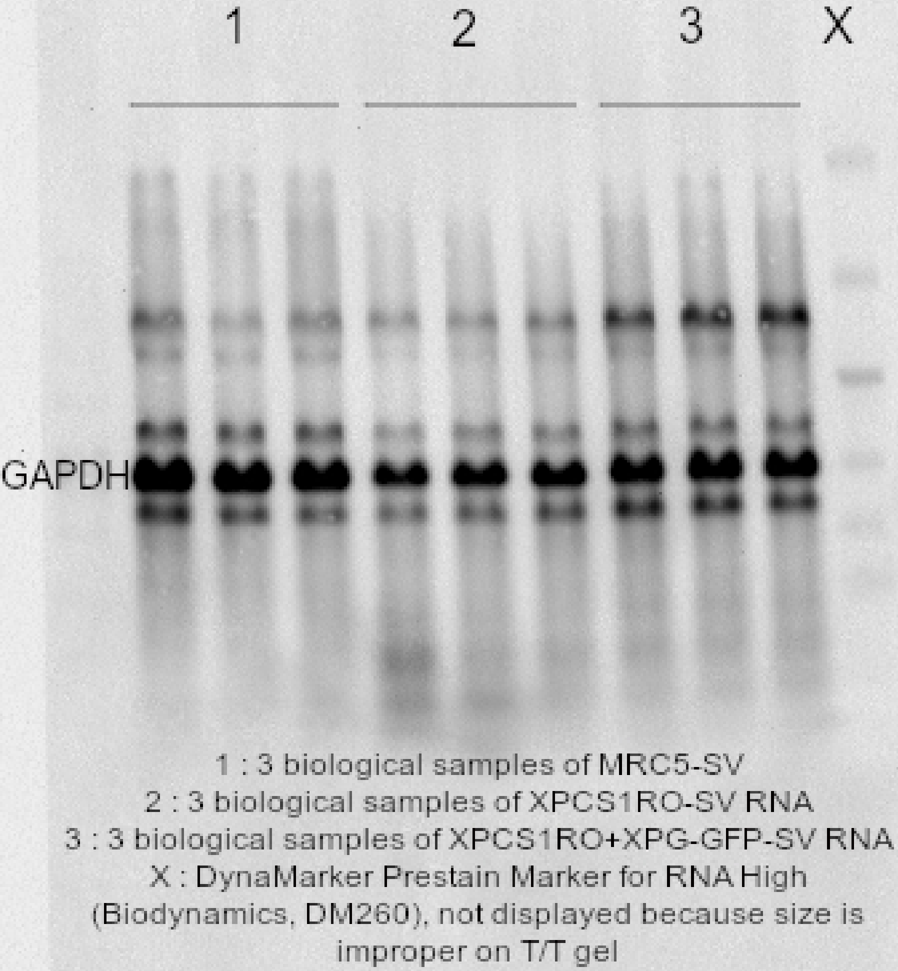

1 : 3 biological samples of MRC5-SV
2 : 3 biological samples of XPCS1RO-SV RNA
3 : 3 biological samples of XPCS1RO+XPG-GFP-SV RNA
X : DynaMarker Prestain Marker for RNA High (Biodynamics, DM260), not displayed because size is improper on T/T gel

GAPDH band in Figure 4B and C

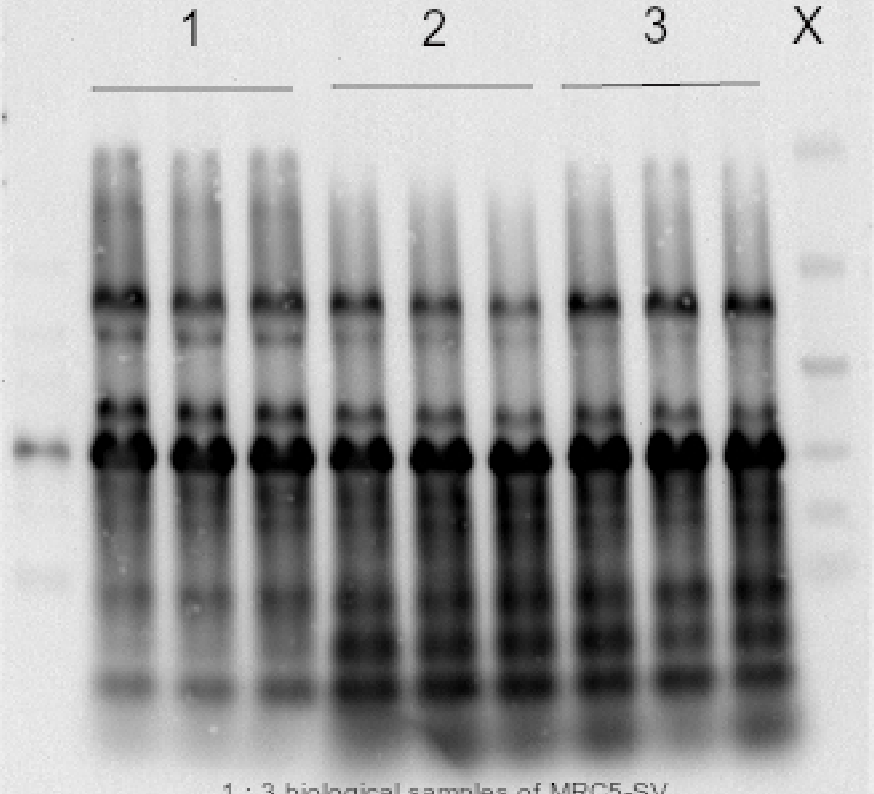

1 : 3 biological samples of MRC5-SV
2 : 3 biological samples of XPCS1RO-SV RNA
3 : 3 biological samples of XPCS1RO+XPG-GFP-SV RNA
X : DynaMarker Prestain Marker for RNA High (Biodynamics, DM260), not displayed because size is improper on T/T gel

rRNA bands on figure 4B

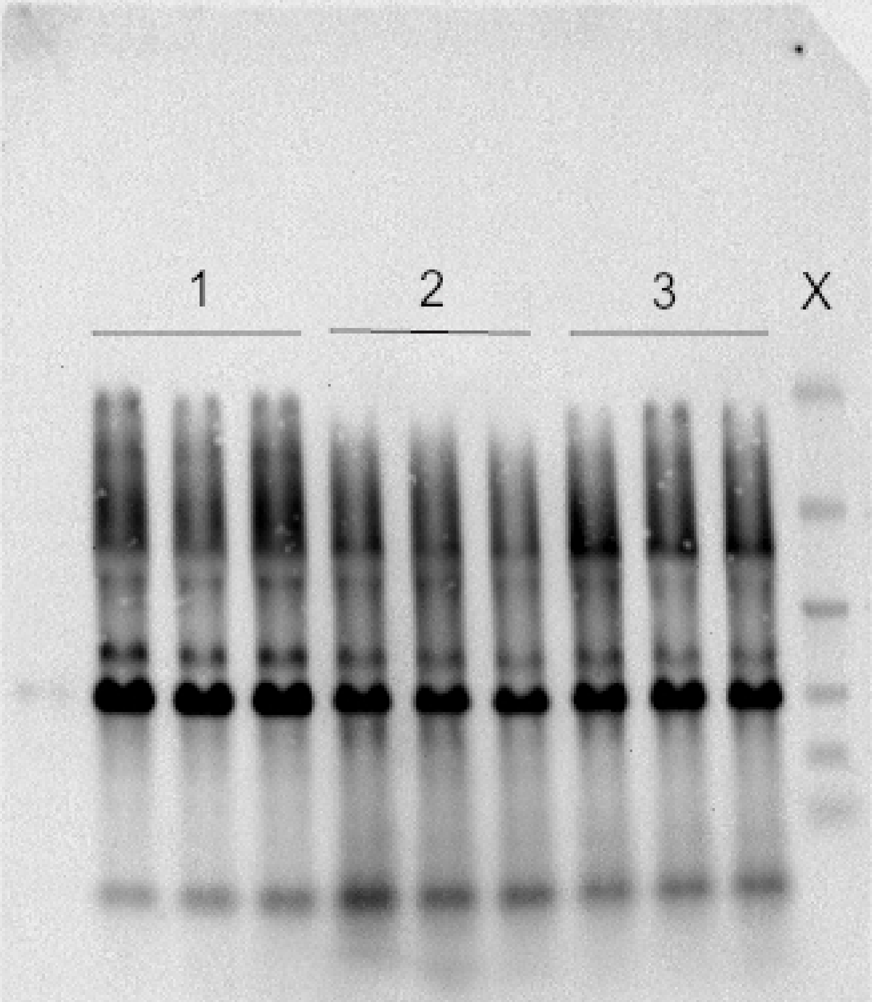

1 : 3 biological samples of MRC5-SV
2 : 3 biological samples of XPCS1RO-SV RNA
3 : 3 biological samples of XPCS1RO+XPG-GFP-SV RNA
X : DynaMarker Prestain Marker for RNA High (Biodynamics, DM260), not displayed because size is improper on T/T gel

rRNA bands in figure 4C

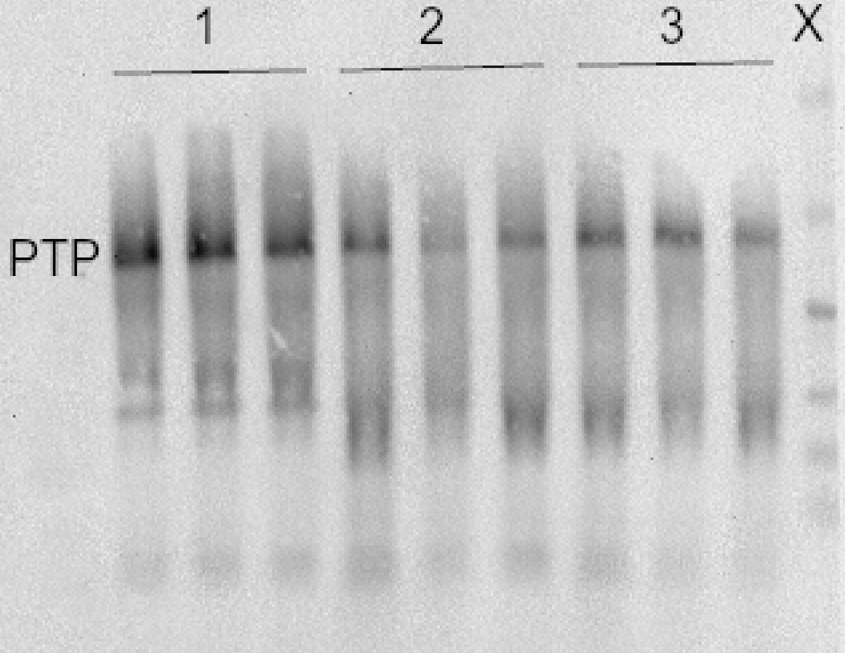

1 : 3 biological samples of MRC5-SV
2 : 3 biological samples of XPCS1RO-SV RNA
3 : 3 biological samples of XPCS1RO+XPG-GFP-SV RNA
X : DynaMarker Prestain Marker for RNA High (Biodynamics, DM260), not displayed because size is improper on T/T gel

PTP band in Figure 4D

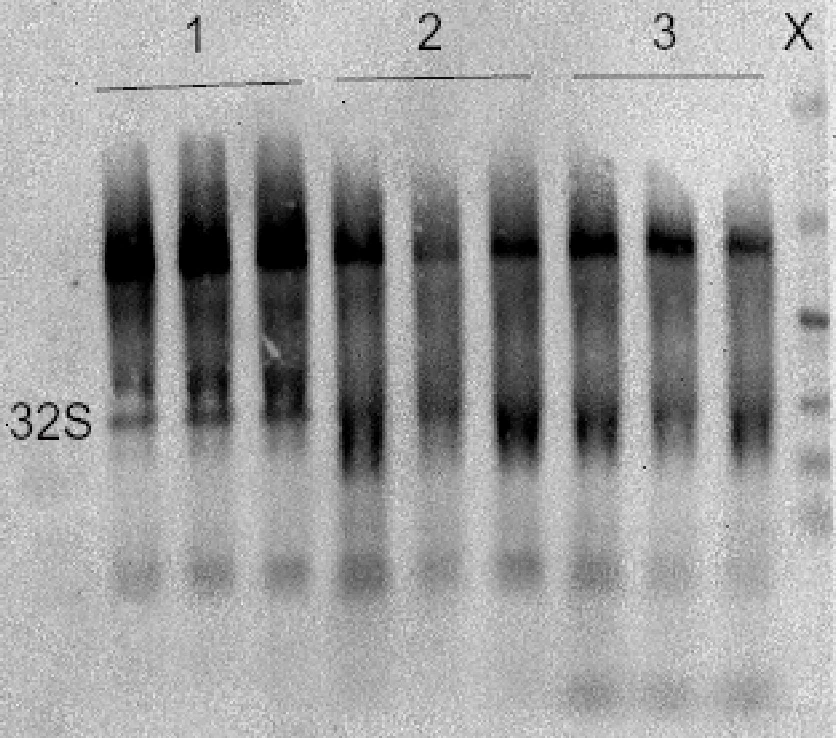

1 : 3 biological samples of MRC5-SV
2 : 3 biological samples of XPCS1RO-SV RNA
3 : 3 biological samples of XPCS1RO+XPG-GFP-SV RNA
X : DynaMarker Prestain Marker for RNA High (Biodynamics, DM260), not displayed because size is improper on T/T gel

32S rRNA band in Figure 4D

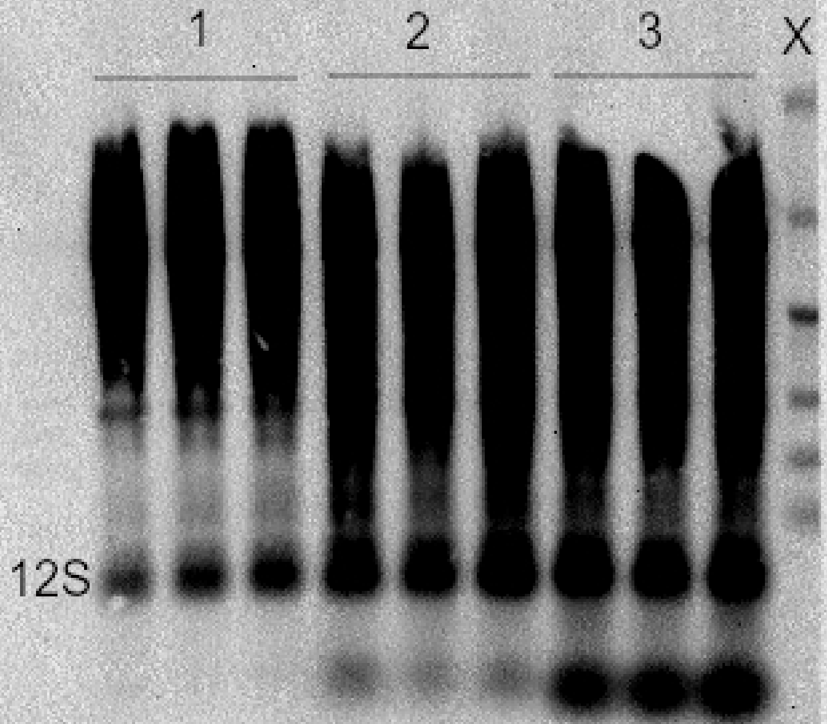

1 : 3 biological samples of MRC5-SV
2 : 3 biological samples of XPCS1RO-SV RNA
3 : 3 biological samples of XPCS1RO+XPG-GFP-SV RNA
X : DynaMarker Prestain Marker for RNA High (Biodynamics, DM260), not displayed because size is improper on T/T gel

12S band used for quantification
